# Ocular inflammation after agitation of siliconized and silicone oil-free syringes: a randomized, double-blind, controlled clinical trial

**DOI:** 10.1186/s40942-022-00387-z

**Published:** 2022-06-17

**Authors:** Natasha Ferreira Santos da Cruz, Murilo Ubukata Polizelli, Felipe Picanço Muralha, Clarice Neuenschwander Lins de Morais, Octaviano Magalhães Silva Junior, Mauricio Maia, Gustavo Barreto Melo, Michel Eid Farah

**Affiliations:** 1 Department of Ophthalmology, Federal University of São Paulo, São Paulo, SP Brazil; 2 Department of Virology, Foundation Oswaldo Cruz, Aggeu Magalhães Institute, Recife, PE Brazil; 3 Hospital de Olhos de Sergipe, Rua Campo do Brito, 995, São José, Aracaju, SE 49020-380 Brazil

**Keywords:** Intravitreal injection, Silicone Oil, Syringe

## Abstract

**Background:**

Noninfectious endophthalmitis may be misdiagnosed, leading to serious clinical implications. So far, its causative factors remain unknown. Therefore, this study assessed the role of silicone oil and syringe agitation in the development of inflammation after intravitreal injection of aflibercept.

**Methods:**

A randomized, double-blind, controlled clinical trial included subjects with an indication of intravitreal antiangiogenic therapy prior to vitrectomy for proliferative diabetic retinopathy. Aflibercept was injected 48 h before surgery. The control group received the injection without agitation, while the intervention group was injected with a previously agitated syringe by flicking with either a siliconized or silicone oil-free syringe. The primary endpoint was the presence of anterior chamber reaction (ACR) at 48 h. Aqueous samples were collected and underwent cytometric bead array analysis for quantification of interleukins and chemokines.

**Results:**

Forty-one individuals were included (21 in the agitation group and 20 in the no-agitation group). None of the included eyes showed baseline signs of AC cells, hyperemia or pain complaint, while 10% of control group and 80% of agitation group showed AC cells 48 h after injection of aflibercept with SR syringe. There were no differences in the mean variations of all cytokines and chemokines by agitation status. However, there was a marginally significant increase between the mean variations of IP-10 (p = 0.057) and IL-8 (p = 0.058) in the siliconized one.

**Conclusion:**

This clinical trial discloses a potential role of agitation and siliconized syringes in the development of inflammation after an intravitreal injection of aflibercept. These findings have important clinical implications for all healthcare practitioners who perform intravitreal injections.

*Trial Registration:* Brazilian Registry of Clinical Trials, RBR-95ddhp. Registered 12 May 2019, http://www.ensaiosclinicos.gov.br/rg/RBR-95ddhp/

## Background

Ocular angiogenesis is a cause of severe visual loss and ocular morbidity worldwide. However, the development of biologics targeting vascular endothelial growth factor (anti-VEGF) has revolutionized the treatment of a plethora of retinal angiogenic disease processes [1]. In 2019, Avastin (bevacizumab) was ranked 5th in terms of top global sales of pharmaceutical products, emphasizing the impact of anti-angiogenics in medicine [2]. In ophthalmology, intravitreal injections (IVIs) surpassed cataract surgery as the most commonly performed intraocular treatment worldwide [3].

Most healthcare providers are unaware that most commercially available syringes worldwide were not developed or approved specifically for ophthalmic use. For instance, only recently has the scientific community paid attention to the silicone oil (SO) droplets that are released by syringes and found in the vitreous of patients [4, 5]. These droplets could lead to the complaint of annoying floaters that, in some cases, require vitrectomy [6].

Additionally, some studies have also shown that some medications are more likely to cause eye inflammation than others [6–8]. However, the causes are uncertain. Our group reported a case–control study that associated inflammation after IVI of aflibercept with the use of a specific brand of syringe (SR—Saldanha Rodrigues, Manaus-Brazil) [9]. Likewise, significant changes in agitation of the syringe to minimize the entry of air into the eye may lead not only the release of SO but also to protein aggregation [10–12].

Although some studies have proven that the needles may also contain small amounts of SO, this appears to be a less important source of SO microdroplets [13].

In view of so many factors that can influence IVI and the onset of inflammation in the postoperative period, this study was carried out to assess the role of agitation of both a siliconized and a silicone-free syringe in the development of inflammation after IVI of aflibercept.

## Methods

### Patients and groups

A randomized, double-blind, controlled clinical trial included subjects with an indication for aflibercept 48 h prior to vitrectomy for proliferative diabetic retinopathy. The study was divided into 4 groups, differing according to handling technique (flicking vs. no flicking) and type of syringe (siliconized vs. SO-free). Group 1 consisted of 10 eyes of 10 patients, in which the siliconized SR (Saldanha-Rodrigues, 1 mL, Brazil) syringe was used for the injection without any agitation (SR–NA); group 2 consisted of 10 eyes of 10 patients, in which the SR syringe was used for the injection with agitation (SR–A); group 3 consisted of 10 eyes of 10 patients, in which the oil-free HSW (HSW Norm- Ject, 1 ml, Germany) syringe was used for the injection without agitation (HSW–NA); group 4 consisted of 11 eyes of 11 patients, in which the HSW syringe was used for the injection with agitation (HSW–A).

Research coordinators randomly assigned eligible patients using the Randomizer for clinical trials application. Following eligibility screening, the system generated a unique number. The research coordinator communicated the retina specialist who would handle the syringe and reported the number. The randomization schedule was stratified by site in 4 blocks. Allocation only occurred once the patient received the injection. Study investigators, research coordinators, patient care teams and the patients were blinded to treatment allocation.

The study was approved by the institution's ethics committee (CAAE: 12806619.6.0000.5505) and by the WHO international clinical trials registry platform (UTN: U1111-1238-9497; RBR-95ddhp) and was performed at the Ophthalmology Department of the Federal University of São Paulo. Informed consent was obtained and the research adhered to the tenets of the Declaration of Helsinki.

### Sample collection

All injections were performed in a standardized fashion. Briefly, a topical anesthetic pre-injection and povidone iodine were instilled, a lid speculum was put in place, and aflibercept was drawn from the vial. In the agitation group, the surgeon flicked the syringe in a standardized way (with the same intensity and number of taps—five, keeping the tip of the needle in a vertical position and oriented downwards). In the control group, the needle was directed downwards with gentle movements, but without any agitation. In both groups, syringe priming (complete movement of the plunger before aspirating the medication) was not allowed. The injections were administered 3.5 mm from the limbus. Antibiotic drops were used. The physician who prepared the medication was not the same who injected it. Both syringes were from the same lot.

Immediately after injection of the medication, undiluted aqueous humor samples (50–100 μL) were obtained through a limbal paracentesis site using a 30G needle with a tuberculin syringe (1 mL). At 48 h after the injection, before the surgery itself, the same procedure was performed.

Both aqueous samples were immediately frozen after the procedure and stored in a sterilized plastic container (Corning Inc., Troy, MI, USA) at − 80 °C in the dark until assays were performed. All samples were assayed within 6 months of collection.

### Inclusion and exclusion criteria

**Fig. 1:**
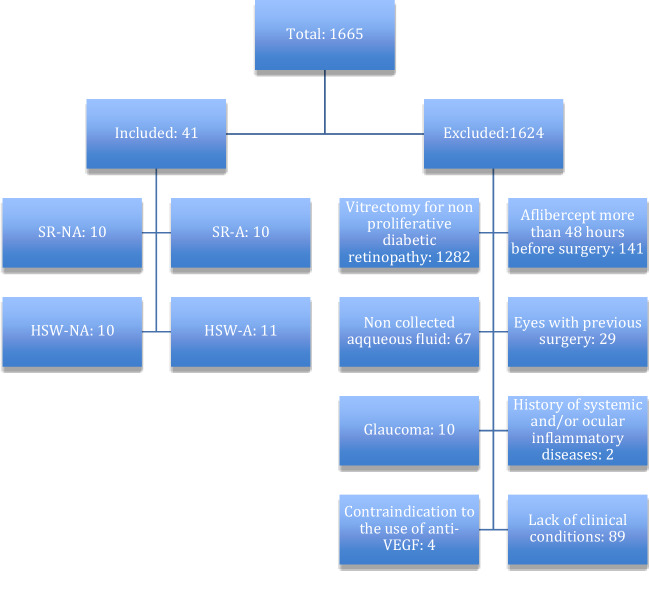
CONSORT flow diagram showing enrolled patients in the clinical trial

Among 1665 patients who underwent vitrectomy in our department from July 2019 to February 2020, we selected those who met our eligibility criteria (Fig. 1). Inclusion criteria were, as follows: (1) vitrectomies performed for proliferative diabetic retinopathy; (2) having performed aflibercept injection 48 h before surgery; (3) aqueous fluid collected 48 h before surgery and at the beginning of surgery; and (4) surgery-naïve eyes. Exclusion criteria were as follows: (1) glaucoma; (2) previous vitrectomy surgery; (3) history of systemic and/or ocular inflammatory diseases; (4) patients with contraindication to the use of anti-VEGF and (5) postponement of surgery due to lack of clinical conditions. Therefore, a total of forty-one patients (41 eyes) satisfied our eligibility criteria.

### Clinical evaluation of inflammation

A retina specialist (M.P.) who did not have access to randomization, to the handling technique or type of syringe, evaluated the presence of anterior chamber inflammatory reaction (ACR) immediately before injection and 48 h later, immediately before vitrectomy. The international classification of uveitis was used to measure inflammation [14]. Other data were also analyzed in these assessments: complaints of pain, conjunctival hyperemia/ciliary injection and intraocular pressure (IOP).

### Measurement of cytokines and chemokines via Cytometric Bead Assay

The levels of cytokines and chemokines in the aqueous humor were quantified using the Cytometric Bead Assay (CBA) method, following the manufacturer's instructions (Becton–Dickinson/BD Biosciences, USA). The following cytokines were evaluated: interleukin 6 (IL-6), IL-1 beta, IL-8, IL-10, TNF and IL-12p70, using the Human Inflammatory Cytokine kit (BD). Also, the following chemokines were evaluated: CXCLB/IL-8, CCL5/RANTES, CXCL9/MIG, CCL2/MCP -1 and CXCL10/IP-10, using the Chemokine Kit (BD).

First, 25 μL of capture beads, labeled with monoclonal anti-cytokine antibodies with different fluorescence intensities, were transferred to tubes for testing and negative control. Next, 25 μL of patient samples were added to their respective tubes, followed by incubation for 1 h at room temperature with protection from light. Subsequently, 25 µL of detection reagent containing phycoerythrin (PE)-conjugated anti-cytokine antibodies were added. The mixtures were incubated for 2 h at room temperature with protection from light. Next, the spheres were washed with 0.5 mL of washing solution and centrifuged at 200xg for 5 min. Carefully, the supernatant was discarded by inverting the tube and 300 µL of buffer solution were added to resuspend the beads. The beads were acquired within 24 h using the FACScalibur Flow Cytometer (BD). Analyses were performed using the FCAP Array software, version 3.01 (BD). A standard curve was drawn using a serial dilution of the cytokine standards present in the kits.

### Statistical analysis

The sample size calculation was based on the formula proposed by Sakpal [15], considering a significance level of 5%, 90% power, two-sided test, with a difference of 20% in one group and 80% in the other. Thus, the ideal sample would be 10 participants in each group, totaling 40 participants.

The SPSS version 20.0 and STATA 12 were used for the analyses. Chi-squared, Fisher´s exact test, independent t tests, ANOVA, Mann–Whitney and Kruskal–Wallis test evaluated differences in characteristics between groups. The Kolmogorov–Smirnov test investigated the distributional characteristics of study-dependent variables. To evaluate the behavior of cytokines and chemokines by time and evaluation and conditions of IVI, Generalized estimating equations (GEE) models with identity linkage function and normal marginal distribution were used. The GEE approach considered within-person variability and accounted for correlated data resulting from repeated measurements across different time points and multiple observations of the same individual. The level of significance was set at 0.05, two-tailed.

## Results

Table 1 shows the four intervention groups. Distinct distributions of postoperative hyperemia (p = 0.032) and insulin dependence (p = 0.038) were observed. Thus, the SR-A group showed the highest percentage of hyperemia in the postoperative period (30.0 versus 0%) and the absence of insulin- dependent patients (60.0 versus a maximum of 45.5%) compared to the other groups. However, despite the difference regarding the use of insulin, there are no studies associating these two measures, which only reflects that randomization cannot reach all criteria.

**Table 1:** Demographic and clinical characteristics of patients

	Total (N=41)	Group				p^ǂ^	p^SP^	p^CP^	p^SR^	p^HSW^
		SR-NA (N=10, 24.4%)	HSW-NA (N=10, 24.4%)	SR-A (N=10, 24.4%)	HSW-A (N=11, 26,8%)		HSW x NA	HSW x NA	CP x A	CP x A
Gender, n (%)						0.109	0.628	0.198	0.628	0.198
Female	18 (43.9)	2 (20.0)	4 (40.0)	4 (40.0)	8 (72.7)					
Male	23 (56.1)	8 (80.0)	6 (60.0)	6 (60.0)	3 (27.3)					
Laterality, n (%)						0.183	0.650	0.080	0.350	0.198
OD	24 (58.5)	5 (50.0)	7 (70.0)	8 (80.0)	4 (36.4)					
OS	17 (41.5)	5 (50.0)	3 (30.0)	2 (20.0)	7 (63.6)					
Pain. n (%)						–	–	–	–	–
No	41 (100.0)	10 (100.0)	10 (100.0)	10 (100.0)	11 (100.0)					
VA (Pre/Post op), n (%)						0.997	1.000	1.000	0.812	1.000
20/100	1 (2.4)	0 (0.0)	0 (0.0)	0 (0.0)	1 (9.1)					
20/100P	1 (2.4)	1 (10.0)	0 (0.0)	0 (0.0)	0 (0.0)					
20/200	2 (4.9)	1 (10.0)	0 (0.0)	0 (0.0)	1 (9.1)					
20/80	1 (2.4)	0 (0.0)	1 (10.0)	0 (0.0)	0 (0.0)					
CF 0.5M	4 (9.8)	1 (10.0)	1 (10.0)	1 (10.0)	1 (9.1)					
CF 1.0M	5 (12.2)	1 (10.0)	1 (10.0)	2 (20)	1 (9.1)					
CF 1.5M	1 (2.4)	0 (0.0)	1 (10.0)	0 (0.0)	0 (0.0)					
CF 2.0M	1 (2.4)	0 (0.0)	1 (10.0)	0 (0.0)	0 (0.0)					
HM	23 (56.1)	5 (50.0)	5 (50.0)	7 (70.0)	6 (54.5)					
LP	2 (4.9)	1 (10.0)	0 (0.0)	0 (0.0)	1 (9.1)					
Hyperemia—pre, n (%)						–	–	–	–	–
No	41 (100.0)	10 (100.0)	10 (100.0)	10 (100.0)	11 (100.0)					
Hyperemia—post, n (%)						0.032	–	0.090	0.211	–
Yes	3 (7.3)	0 (0.0)	0 (0.0)	3 (30.0)	0 (0.0)					
No	38 (92.7)	10 (100.0)	10 (100.0)	7 (70.0)	11 (100.0)					
Phakic/Pseudophakic, n (%)						0.366	0.211	1.000	1.000	0.635
Phakic	35 (85.4)	10 (100.0)	7 (70.0)	9 (90.0)	9 (81.8)					
Pseudophakic	6 (14.6)	0 (0.0)	3 (30.0)	1 (10.0)	2 (18.2)					
Type of DM, n (%)						0.360	1.000	0.214	1.000	0.586
DM 1	5 (12.2)	1 (10.0)	1 (10.0)	0 (0.0)	3 (27.3)					
DM 2	36 (87.8)	9 (90.0)	9 (90.0)	10 (100.0)	8 (72.7)					
Insulin dependent. n (%)						0.038	1.000	0.670	0.057	0.149
Yes	28 (68.3)	9 (90.0)	9 (90.0)	4 (40.0)	6 (54.5)					
No	13 (31.7)	1 (10.0)	1 (10.0)	6 (60.0)	5 (45.5)					
A/NA, n (%)						–	–	–	–	–
NA (non agitação)	20 (48.8)	10 (100.0)	10 (100.0)	0 (0.0)	0 (0.0)					
A (agitation)	21 (51.2)	0 (0.0)	0 (0.0)	10 (100.0)	11 (100.0)					
SR/HSW, n (%)						–	–	–	–	–
SR	20 (48.8)	10 (100.0)	0 (0.0)	10 (100.0)	0 (0.0)					
HSW	21 (51.2)	0 (0.0)	10 (100.0)	0 (0.0)	11 (100.0)					
IOP pre						0.221^a^	0.145^a^	0.325^a^	0.657^a^	0.039^a^
Average (SD)	14.71 (3.32)	14.20 (3.65)	16.40 (2.76)	14.90 (3.28)	13.45 (3.27)					
Median (Min–Max)	15.00 (8.00–21.00)	13.50 (8.00–20.00)	16.00 (12.00–21.00)	15.50 (10.00–20.00)	14.00 (8.00–18.00)					
N	41	10	10	10	11					
IOP post						0.581^a^	0.450^a^	0.468^a^	0.594^a^	0.437^a^
Average (SD)	13.71 (3.78)	13.60 (3.78)	14.90 (3.75)	12.50 (5.17)	13.82 (2.14)					
Median (Min–Max)	13.00 (4.00–20.00)	12.50 (8.00–19.00)	15.00 (10.00–20.00)	12.50 (4.00–20.00)	13.00 (10.00–18.00)					
N	41	10	10	10	11					
Blood glucose						0.254^a^	0.339^a^	0.230^a^	0.308^a^	0.210^a^
Average (SD)	154.86 (68.24)	197.00 (112.03)	153.22 (54.28)	150.80 (51.67)	126.70 (33.13)					
Median (Min–Max)	141.00 (83.00–353.00)	175.00 (84.00–353.00)	141.00 (86.00–259.00)	139.00 (85.00–242.00)	138.00 (83.00–167.00)					
N	37	8	9	10	10					

p^ǂ^—descriptive level of Fisher's exact test, ANOVA(^a^) or Kruskal-Wallis (^b^).

p^SP^, p^CP^, p^SR^, p^HSW^—descriptive levels of Fisher's exact test, Student’s t (^a^) or Mann-Whitney (^b^) test

(A) e (B) show different means according to Dunn-Bonferroni multiple comparisons when comparing the four groups

**Fig. 2:**
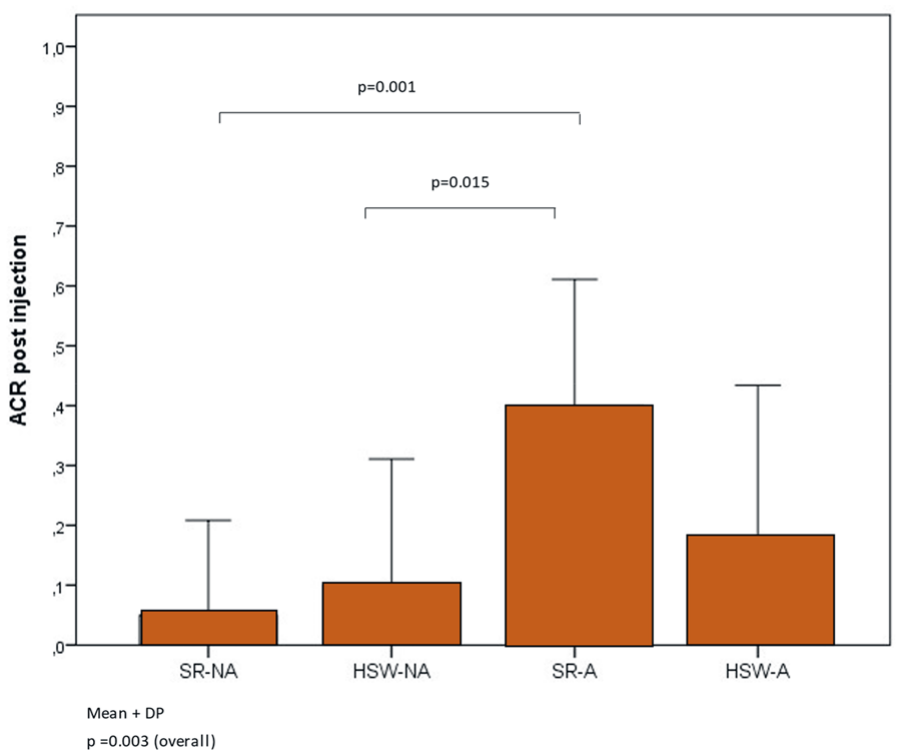
Average of post injection anterior chamber reaction (ACR) per group

**Fig. 3:**
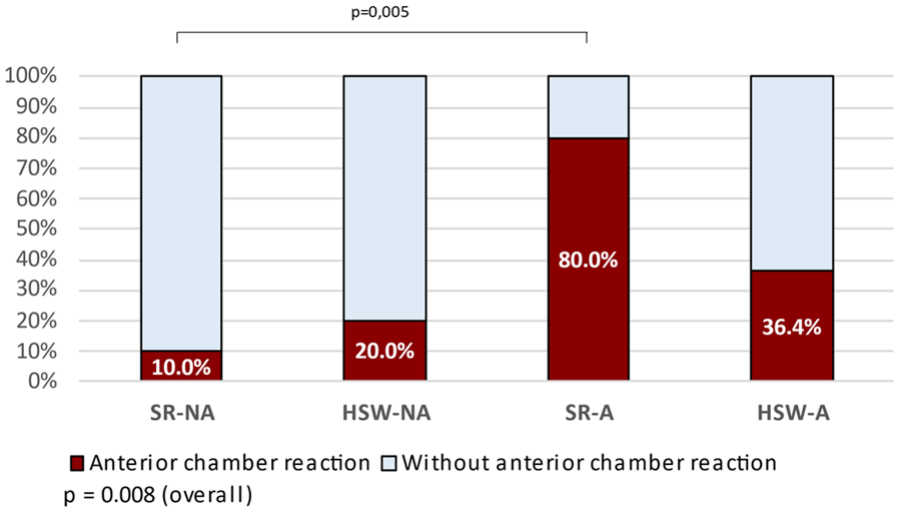
Distribution of post injection anterior chamber reaction (ACR)—classification by group

According to Figs. 2 and 3, considering the four intervention groups, means (p = 0.003) and different distributions (p = 0.008) of anterior chamber inflammatory reaction were found after the injection. The SR-A group had a higher mean anterior chamber inflammatory reaction post injection than the other groups (1.1 ± 0.97 cells vs. 0.05 ± 0.16 in SR-NA, 0.41 ± 0.66 in HSW-A, and 0.15 ± 0.34 in HSW-NA). Additionally, it was found that the SR-A had a higher percentage (80.0%) of inflammation than the SR-NA group (10.0%). It is notable that 15 patients had inflammation after the injection and 12 (80.0%) of them were in the agitation group.

Analysis of cytokines and chemokines was initially carried out to detect the influence of agitation without considering the types of syringes. It was found that there were no differences in the mean variations of all cytokines and chemokines by agitation status. Nevertheless, it was observed that there was a marginally significant difference between the mean variations of IP-10 (p = 0.097) and MCP-1 (p = 0.056). This means that with agitation, there was a mean increase in IP-10 (p = 0.001) and MPC-1 (p = 0.001) after the intervention, as opposed to the condition with no agitation, in which no differences were observed (data not shown). That is, although these markers increased with agitation, there was no statistical significance, because of the large variations.

Subsequently, cytokines and chemokines by time of evaluation and agitation condition were analyzed separately according to each type of syringe. As shown in Table 2, there were no differences in the mean variations of all cytokines and chemokines by agitation status in the SR syringe. However, there was a marginally significant difference between the mean variations of IP-10 (p = 0.057) and IL-8 (p = 0.058). Thus, in the agitated condition, there was a mean increase in IP-10 (p = 0.003) and IL-8 (p < 0.001) after the intervention, as opposed to the condition without agitation, in which no differences were observed between evaluation times. Regarding the HSW syringe, there were no differences in the mean variations of all cytokines and chemokines by agitation status in the HSW syringe (data not shown).

**Table 2:** Summary measurements of cytokines and chemokines by evaluation moments and agitation condition in the SR syringe

				p-value		
	PE	PO	PO-PE	Group	Time	TimexGroup
Cytokines						
IL-12p70(pg/mL)				0.668	0.880	0.170
SP	0.132 (0.279)	0.000 (0.000)	− 0.132 (0.279)		0.057^ǂ^	
CP	0.092 (0.237)	0.077 (0.243)	− 0.015 (0.055)		<0.001^ǂ^	
TNF (pg/mL)				0.995	0.023	0.579
SP	0.368 (0.581)	0.064 (0.138)	− 0.304 (0.579)		0.044^ǂ^	
CP	0.369 (0.264)	0.169 (0.303)	− 0.200 (0.235)		0.263^ǂ^	
IL-10 (pg/mL)				0.631	0.969	0.872
SP	0.143 (0.226)	0.166 (0.254)	0.023 (0.330)		1.000^ǂ^	
CP	0.209 (0.475)	0.211 (0.281)	0.002 (0.283)		1.000^ǂ^	
IL-6 (pg/mL)				0.417	0.013	0.085
SP	25,815 (42,437)	936,687 (1.412,46)	910.872 (1.409,668)		1,000^ǂ^	
CP	2.120,673 (6,649,132)	7.742,183 (10,103,569)	5.621,51 (9,013,125)		0.007^ǂ^	
IL-1β (pg/mL)				0.346	0.330	0.903
SP	0.174 (0.291)	0.069 (0.155)	− 0.105 (0.344)		0.512^ǂ^	
CP	0.089 (0.268)	0.000 (0.000)	− 0.089 (0.268)		0.671^ǂ^	
IL-8 (pg/mL)				0.891	0.068	0.651
SP	84,172 (124,376)	149,36 (168,312)	65,188 (68,703)		0.396^ǂ^	
CP	93,253 (162,579)	190,866 (163,324)	97,613 (228,698)		0.108^ǂ^	
Chemokines						
IP-10 (pg/mL)				0.593	0.005	0.057
SP	112,071 (77,715)	146,979 (99,204)	34,908 (84,366)		1.000^ǂ^	
CP	176,633 (316,682)	390,071 (457,088)	213,438 (300,935)		0,003^ǂ^	
MCP-1 (pg/mL)				0.861	0.080	0.213
SP	1.023,24 (683,41)	2.243,311 (1.477,744)	1.220,071 (918,231)		1.000^ǂ^	
CP	1.508,242 (2.320,969)	7.588,443 (12.720,342)	6.080,201 (12.976,316)		0.055^ǂ^	
MIG (pg/mL)				0.530	0.138	0.148
SP	133,928 (231,658)	129,173 (202,629)	− 4.755 (47,310)		1.000^ǂ^	
CP	61,951 (88,722)	235,858 (434,639)	173,907 (409,053)		0.093^ǂ^	
RANTES (pg/mL)				0.048	0.653	0.360
SP	0.548 (0,858)	0.837 (0,629)	0.289 (0,818)		1.000^ǂ^	
CP	1.936 (2,905)	1.552 (1,178)	− 0.384 (2,312)		0.921^ǂ^	
IL-8 (pg/mL)				0.568	<0.001	0.058
SP	77,427 (87,428)	134,79 (143,603)	57,363 (89,646)		0.139^ǂ^	
CP	49,779 (57,029)	191,932 (143,606)	142,153 (118,93)		<0.001^ǂ^	

p—descriptive level of the effects of time, group and interaction between group and treatment of the EEG model.

p^ǂ^—descriptive level of post hoc tests for comparison of means between evaluation moments, in each group, with Bonferroni correction.

Average (SD)

Cytokines: n=10 and n=10, respectively for SP and CP. Chemokines: n=10 and n=10, respectively for SP and CP

## Discussion

In the last years, studies of inflammatory mediators were performed to assess diabetic retinopathy progression and responses to specific drugs [16–19]. However, there are currently no published clinical trials addressing handling technique and type of syringe, as well as proinflammatory chemokines and cytokines, on inflammatory reaction after IVIs.

The primary endpoint of this study was the presence of anterior chamber inflammatory reaction at baseline and 48 h later. We found a much higher rate of anterior chamber inflammatory reaction 48 h after the aflibercept IVI with a siliconized syringe. This finding led us to believe that agitation plays a role in inflammation after aflibercept IVI using a siliconized syringe.

Most syringes used for IVIs are coated with SO, which acts as a lubricant to ensure smooth plunger movement and to guarantee the functionality of the syringe throughout the product’s shelf life. However, the SO may follow the intravitreal drug and lead to symptomatic deposition of SO droplets. The use of syringes with low dead space and typical mechanical stress, during shipping and handling has been shown to increase the risk [4, 20–24].

It has already been reported that drugs, such as protein based ones (Insulin, abatacept and other biopharmaceuticals), are highly sensitive to the use of SO-coated syringes [25, 26]. There is a concern about the potential of silicone oil increasing immunogenicity by functioning as an adjuvant or increasing protein aggregation [27–31] and thereby reducing drug safety and efficacy [32].

Factors known to unfold or otherwise denature proteins may also increase their immunogenicity. Some of them are: (1) temperature fluctuation [33] or failure to maintain a proper temperature during both shipping and storage, (2) air bubbles from the vial [34] and (3) agitation stress occurring during end-over-end rotation, as well as flicking of the syringe at the time of injection [21, 35].

Inflammation after an IVI of an antiangiogenic drug is an uncommon but alarming clinical occurrence that might easily be mistaken for infectious endophthalmitis. The American Society of Retina Specialists Therapeutic Surveillance Committee received notifications of 56 cases of aflibercept-related sterile inflammation from December 2011 through February 2014 [6]. While some of these reported cases responded to topical corticosteroid treatment and observation alone, others needed to undergo vitrectomy to treat cases with a severe inflammation. One of the different potential mechanisms involved impurities introduced from the vehicle, such as SO droplets which may lead to toxic shock-like syndrome [36].

In a case–control study, we reported a link between noninfectious inflammatory reactions after IVI of aflibercept in six patients and the use of a specific model of syringe (SR). The observation of particles in the vitreous, the ease of glide of the plunger and a further laboratory study showed that the SR syringes release a significant amount of SO, especially after agitation [9]. Also, many studies have reported an important association of SO-water interfaces (siliconized syringe walls), air–water interfaces (air bubbles), and agitation stress as triggers leading to protein aggregation and particle formation [28, 29, 37]. The highest particle concentrations were found in agitated, siliconized syringes containing an air bubble [29].

Since aflibercept is a fusion protein, we hypothesized that it should be more prone to yield insoluble and protein aggregates than other anti-VEGF antibodies. Considering that flicking the syringe might cause an increase in some inflammatory chemokines and that SO droplets were also reported to act as immunologic adjuvants, the inflammatory reaction observed in the current patients might have been caused by those interactions [9, 11, 38].

Increased secretion of macrophage inflammatory protein-1 alpha (MIP-1a), IL-6, IL-8, and tumor necrosis factor (TNF) from human peripheral blood mononuclear cells (hPBMCs) of a small donor panel was observed in response to the pharmaceutical protein solution containing SO droplets. Additionally, increase of anti-drug antibody in response to SO-containing samples compared to the absence of SO has been shown. Therefore, the use of SO-free syringes could contribute to the enhanced safety of reconstituted biopharmaceuticals [39].

We observed that the levels of proinflammatory factors, such as IP-10, proangiogenic cytokines MCP1 and IL-8, increased marginally in the aqueous humor of patients after flicking the syringe. IP-10 activates a Th1 cell-mediated immune response [40]. This chemokine promotes inflammation, regulation of cell growth, and angiogenesis in infectious and inflammatory diseases [41, 42].

MCP-1 recruits inflammatory and activated monocytes to inflamed tissues [43]. MCP-1 was detected in high concentration in patients with previous active uveitis. Clinical features of uveitis, such as hyperemia and vasodilation, may be caused by histamine release from mast cells and basophils, induced by the action of MCP-1 [44]. Alternatively, it is possible that subjects with high aqueous humor concentrations of MCP-1 may be prone to developing chronic anterior uveitis [45]. Considering that many patients receive IVIs on a monthly basis, this would be an important marker indicating that syringe flicking could lead to chronic uveitis.

IL-8 it is known as a neutrophil chemotactic factor and T‑cell activator in the innate immune system [46]. Wu et al. [47] reported that recent anti-VEGF injection did not significantly affect IL-8, but aqueous IL-8 was correlated with a worse visual prognosis probably because of its role in fibrous proliferation. Thus, these inflammatory markers seem to demonstrate some role in the inflammatory process after IVIs with syringe agitation.

Although the current study reported multiple findings of interest, there are some limitations worth acknowledging. First, slit-lamp grading cells is subjective, and the inflammatory cells detected by the anterior chamber reaction do not necessarily represent clinically significant inflammation. Second, the use of aqueous humor cytokine assessment may not completely reflect changes in the posterior segment. However, several studies have shown a correlation between vitreous and aqueous humor cytokine levels [48, 49]. As with most of the reports in the literature evaluating aqueous cytokine data, this study was limited by available sample size, especially in subgroup assessment. In addition, the CBA is limited if the cytokine levels are very low, so in this case, some molecules were not included in the statistical analysis [50]. Furthermore, our aqueous humor cytokine concentration measures may not be applicable to other anti-VEGF agents and syringes, as only one medication and two syringe types were evaluated. Finally, it is not appropriate to assume that a particular cytokine plays a role in pathogenesis based simply on measurement of elevated aqueous levels. The release of a particular cytokine could be a result of the whole process, and not necessarily be the cause. Strengths of this study include a single treating physician and a single masked examiner grading inflammation according to a published standard schema. This study design allows for the most accurate direct comparison of differences of the syringes and handling technique.

## Conclusions

This clinical trial revealed a potential role of agitation in the development of inflammation after an IVI of aflibercept. Thus, considering the chronic nature of retinal neovascular diseases, the repeated injections administered throughout the life of the disease and the confined nature of the vitreous, the clinical consequences of the injection of particulate material can be cumulative. Therefore, silicone oil-free syringes should be the standard of care for all intravitreal anti-VEGF injections performed worldwide.

## Data Availability

The datasets used and/or analysed during the current study are available from the corresponding author on reasonable request.

## Abbreviations

A: Agitation
ACR: Anterior chamber inflammatory reaction
Anti-VEGF: Vascular endothelial growth factor
BD: Becton–Dickinson
CBA: Cytometric bead assay
GEE: Generalized estimating equations
hPBMCs: Human peripheral blood mononuclear cells
HSW: HSW Norm-Ject
IOP: Intraocular pressure
IVIs: Intravitreal injections
NA: Non-agitation
OD: Right eye
OS: Left eye
PE: Pre operative
PE: Phycoerythrin
PO: Pos operative
SD: Standard deviation
SO: Silicone oil
SR: Saldanha Rodrigues
TNF: Tumor necrosis factor
VA: Visual acuity
